# Supplementary material for: Sex differences in brain-behavior relationships in the first two years of life
Source: bioRxiv. 2024 Feb 1:2024.01.31.578147. Preprint. [Version 1] doi: 10.1101/2024.01.31.578147 (PMC10862872; doi:10.1101/2024.01.31.578147)
Supplement: Supplement 1 [file media-1.pdf]

**Supplemental Figure 1**

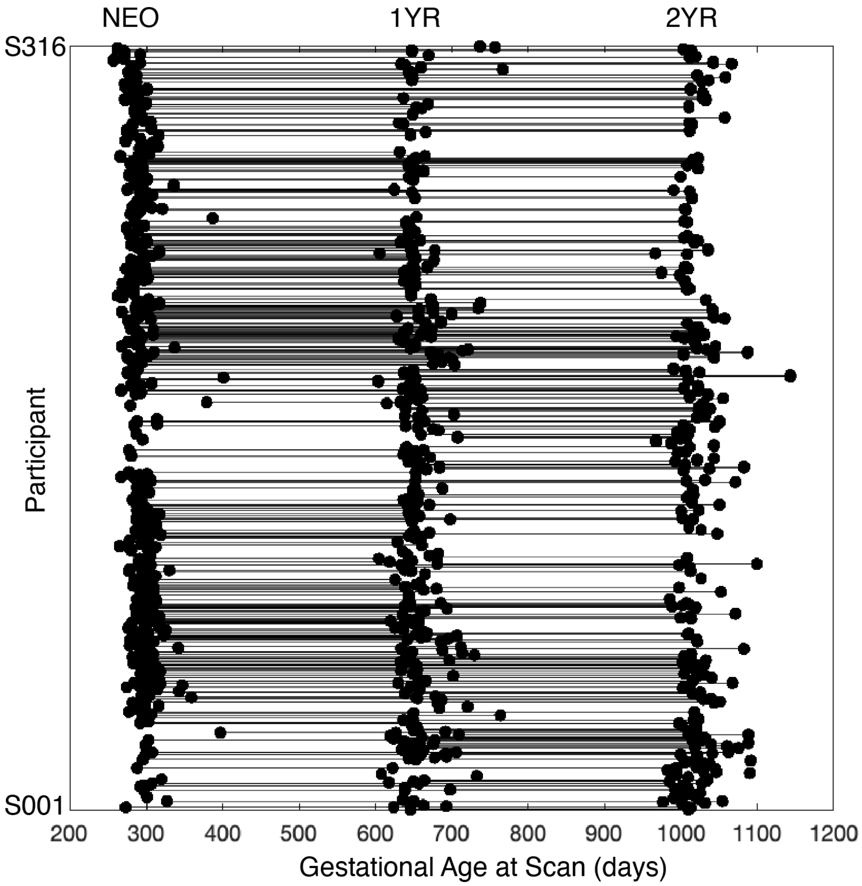


Figure S1. Data distribution. The distribution for all infant datasets with images passing quality control across gestational age at scan (*N*=316, with a total of 484 datasets). rsfMRI scans are represented at each dot and their longitudinal trajectories are represented by each line. Neonates (NEO): *n*=229; 1-year-olds (1YR): *n*=146; 2-year-olds (2YR): *n*=109; Neo and 1YR: *n*=85; 1YR and 2YR: *n*=60; NEO and 2YR: *n*=62; NEO, 1YR, and 2YR: *n*=38.
